# Juice from *Fructus Rosae Roxburghii* normalizes blood lipids in mice with diet‐induced hyperlipidemia*^†^

**DOI:** 10.1002/fsn3.1897

**Published:** 2020-09-18

**Authors:** Pingping Song, Xiangchun Shen

**Affiliations:** ^1^ The State Key Laboratory of Functions and Applications of Medicinal Plants Guizhou Medical University Guiyang China; ^2^ Key Laboratory of Optimal Utilization of Natural Medicine Resources School of Pharmaceutical Sciences Guizhou Medical University Guiyang China

**Keywords:** blood lipids, *Fructus Rosae Roxburghii*, hyperlipidemia, targeted quantitative proteomic analysis, transcriptomic analysis

## Abstract

*Fructus Rosae Roxburghii* (FRR) as a dietary supplement is considered to possess anti‐atherosclerosis (AS), and hyperlipidemia (HLP) is material basis for AS formation, so the effects and molecular mechanism of FRR on diet‐induced hyperlipidemic mice were explored. In Diet IV2 group, hepatic steatosis was significantly relieved; meanwhile, TC, TG, LDL‐C, HDL‐C, and ASI in serum were regulated to control level. Thirty‐seven DCEG in Diet I, Diet II, and Diet IV2 groups were obtained by RNA‐seq analysis. Relative mRNA levels were further determined by qRT‐PCR, of which 28 genes were matched with those detected by RNA‐seq. Ten DCEP were verified by targeted quantitative proteomic analysis, but expressive patterns of only six proteins were correlated with qRT‐PCR data. These DCEG and DCEP played important roles in regulating the biosynthesis of BAs and steroids, fatty acid metabolism, and LPO production. They might cooperatively regulate the function of HDL or RCT by PPAR signaling pathway under the FRR action. As we know, it is the first time the potential anti‐atherosclerotic mechanism of FRR regulating the blood lipids was explored.

## Graphical abstract

Differentially coexpressed genes and proteins might cooperatively regulate the function of HDL or reverse cholesterol transportation (RCT) by PPAR signaling pathway under the action of FRR juice.

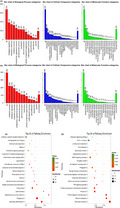

## 1 INTRODUCTION

Hyperlipidemia (HLP) is one of the important risk factors leading to atherosclerosis (AS) (Ross & Harker, 1976), so regulating blood lipids and ameliorating lipid metabolism disorders are considered as important strategies to prevent cardiovascular disease (CVD). HLP induced by lipid metabolism disorders can lead to increase in lipids and lipoproteins in serum (Brischetto, Connor, Connor, & Matarazzo, 1983; Olsson, Pears, McKellar, Mizan, & Raza, 2001), generally characterized by high total cholesterol (TC) or triglyceride (TG), or low high‐density lipoprotein (HDL). These lipoproteins play important role in regulating the blood lipid. Hypertriglyceridemia is a prevalent risk factor for CVD. Elevated TG levels are also important markers for several types of atherogenic lipoproteins (Malloy & Kane, 2001). LDL can transport the cholesterol on the surface of cells by LDL receptor and scavenger receptor, which can prevent excessive lipid accumulation (Goldstein & Brown, 2009; Hansson & Libby, 2006). Damaged LDL receptor would prompt the development of AS by accumulating intracellular lipid. LDL can be transformed into oxidized low‐density lipoprotein (OX‐LDL) under free radical action, which plays a key role for AS formation (Jialal & Devaraj, 1996). HDL can promote the removal of cholesterol from artery walls by reverse cholesterol transportation (RCT). HDL can protect vascular endothelial cells and decrease the intake of smooth muscle cells for LDL, thereby inhibiting the synthesis of glycosaminoglycan (GAGs) and proliferation of smooth muscle cells (Hessler, Robertson, & Chisolm, 1979).

At present, some medicinal and edible plants were considered to regulate blood lipids, such as radix notoginseng powder and ginkgo biloba leaves (Calixto, 2000; Chang et al., 2007). *Fructus Rosae Roxburghii* (the fruit of *Rosa roxiburghii Tratt.f. normalis Rehd. Et Wils*., FRR) belonging to *Rosaceae* family is a special wild resource in Yunnan–Guizhou plateau, which mainly originates from Guizhou Province of China (Li et al., 2016). It has long been considered as healthy fruit improving the digestion, which was recorded for the first time in the Compendium of Materia Medica in 1765 A.D (Liu et al., 2016). Recently, data have shown that FRR has antioxidant, antimutagenic, antiatherogenic, and antitumor effects due to the presence of various phytochemicals, including vitamin C, flavonoids, polyphenol, organic acids, triterpenes, superoxide orgotein dismutase (SOD), and essential oils that have been found in FRR (Burke, Smidt, & Vuong, 2005; Dai & Yang, 2015; Lu & Bao, 2002; Xu, Vidyarthi, Bai, & Pan, 2019). FRR is rich with nutrients and bioactive properties, and can be used as dietary supplement, which can reduce the risk of CVD, especially atherosclerosis (AS) (Zhang et al., 2001). Some experiments have found that FRR juice can significantly reduce intracellular cholesterol and improve antioxidant capacity of LDL in plasma, thus reducing the incidence of AS (Sotherden, Uto‐Kondo, Ayaori, & Ikewaki, 2018).

The anti‐atherosclerosis function of FRR is related to its chemical composition, which may be single function or synergistic effect of bioactive substances, but the mechanisms are still unclear. HLP is the material basis for the formation of AS; at present, we analyzed the effects and molecular mechanism of FRR juice on diet‐induced hyperlipidemic mice. In recent years, RNA‐seq and targeted quantitative protein analysis have become powerful technologies for identification of mRNA and proteins in tissues or cells. The KM mice model with HLP was firstly reproduced; then, expression of key genes and proteins participated in lipid metabolism and related signaling pathways were analyzed in mice, which could explore potential mechanisms of FRR in anti‐atherosclerosis. This not only provides theoretical basis for application of FRR in AS, but also suggests a potential beneficial drug for AS.

## 2 MATERIALS AND METHODS

### 2.1 Materials

FRR from No. 5 Guinong was identified by Key Laboratory of Natural Product Chemistry, Chinese Academy of Sciences. Fresh FRR juice (2 kg) was prepared by automatic squeezer and preserved in fridge (4°C). The male Yunnan KM mice were purchased from Animal Center of Guizhou Medical University (No. SCXK Jing 2012‐0001). The Xuezhikang capsule was used as positive control drug and purchased from Weixin Biotechnology Limited Company (Peking University). All other chemicals and labware were obtained from Kaixin Biotechnology Limited Company.

The mouse feeds were purchased from Xiaoshu Biotechnology Limited Company. The components of normal diet were 45% corn, 20% bean, 20% wheat flour, 10% pear skin, 1% fish meal, 0.2% yeast, 1% salt, 0.58% vegetable oil, 2% bone meal, 0.2% methionine, and 0.02% multivitamin. The high‐fat diet was composed of 78.7% normal diet, 1% cholesterol, 0.3% cholic acid, 10% pork lard, and 10% yolk powder.

### 2.2 The hyperlipidemic mouse model

The healthy mice were divided into four groups with average weight and maintained in different mice cages, with a 12 hr light/dark cycle and free access to purified water. The control mice were fed normal diet (Diet I) starting from 6 weeks of age. The model group was fed high‐fat diet (Diet II). The positive drug group (Diet III) and treatment group (Diet IV) were on high‐fat diet and were fed separately with Xuezhikang capsules or FRR juice. In Diet IV group, 25% (IV1), 50% (IV2), and 100% (IV3) FRR juice were prepared by mixing purified water, and then were given to mice by gavage (0.25 ml/10 g). On day 30, blood was collected by retro‐orbital bleeding; finally, these mice were euthanized by cervical dislocation.

### 2.3 The liver pathology and blood lipids in mice

After mice were anatomized, their livers were immediately weighed and washed by physiological saline (0.9%). For pathological analysis, some hepatic tissues in mice were fixed in 10% neutral formalin, then processed by rinsing, dehydration, wax immersion, embeddedness, HE staining, and sealing, and finally observed by optical microscope. The rests of hepatic tissue were preserved in liquid nitrogen. The collected blood from mice was allowed to clotting at room temperature and then centrifuged at 10 min (500 *g*). The upper serum samples were separated and used for analysis of TC, TG, HDL‐C, and LDL‐C by the BS‐800 automatic biochemical analyzer. The hepatic index was calculated by the ratio of liver weight and body weight. The atherosclerotic index (ASI) is usually used for measurement of AS level, which was calculated by the equation as following: ASI = (TC − HDL)/HDL. If ASI is less than 4, it reflects that AS degree is not serious or in reducing. ASI is smaller, AS degree is much lighter, and the risk of CVD is much lower.

### 2.4 The RNA‐seq analysis for hyperlipidemic mice

RNA extraction. The total RNA of hepatic tissues in mice was extracted by TRIzol reagent in accordance with manufacturer's instructions. The genomic DNA was removed from RNA sample using DNase. The concentration, quality, and integrity of RNA were determined using a NanoDrop 1000 spectrophotometer (Thermo Scientific) and an Agilent 2100 Bioanalyzer (Agilent Technologies). RNA samples with 260/280 nm absorbance ratios of approximately 2.0 and RNA integrity numbers >8.0 were processed for RNA‐seq.

RNA‐seq library construction. The RNA samples from three replicates of nine hepatic tissues of mice fed three experimental diets were prepared 81 libraries. After total RNA was extracted, eukaryotic mRNA was enriched by Oligo (dT) beads, while prokaryotic mRNA was enriched by removing rRNA by Ribo‐ZeroTM Magnetic Kit (Epicentre). Then, enriched mRNA was fragmented into short fragments using fragmentation buffer and reverse transcripted into cDNA with random primers. Second‐strand cDNA was synthesized by DNA polymerase I, in the presence of RNase H, dNTP, and buffer. Then, cDNA fragments were purified with QiaQuick PCR extraction kit, end‐repaired, poly(A)‐added, and ligated to Illumina sequencing adapters. The ligation products were selected by agarose gel electrophoresis, PCR‐amplified, and sequenced using Illumina HiSeqTM 4000 (Gene Denovo). After removing adaptor sequences, ambiguous “N” nucleotides (>5% of “N” ratio), and low‐quality sequences (<10 of quality score), remaining clean reads were assembled using Trinity software. The clean reads were submitted to NCBI Short Read Archive (SRA) database. For homology annotation, non‐redundant sequences were subjected to public databases using BLASTx or BLASTn algorithm (*E*‐value < 10^−5^), including NCBI non‐redundant protein and nucleotide, Gene Ontology (GO), and KEGG.

Bioinformatic Analysis of RNA‐Seq Data. Reads obtained from the sequencing machines were filtered by removing reads containing adapters, reads containing more than 10% of unknown nucleotides (N), and low‐quality reads containing more than 50% of low‐quality (*Q*‐value ≤ 10) bases. The high‐quality clean reads were mapped to ribosome RNA (rRNA) to identify residual rRNA reads. The rRNA removed reads were used for further analysis. The gene abundances were calculated and normalized to RPKM (reads per kb per million reads), which were used for comparing the difference of gene expression. Principal component analysis (PCA) performed with R package models (http://www.r‐project.org/) was used to reveal the structure/relationship of the samples. To identify differentially expressed genes (DEG) between different treatment groups, the edgeR package (http://www.r‐project.org/) was used. The genes with a fold change ≥2 and a false discovery rate (FDR) < 0.05 were identified as DEG, which were then subjected to enrichment analysis of GO functions and KEGG pathways. GO has three ontologies: molecular function, cellular component, and biological process, which were used to describe properties of genes and their products in any organism. All DEG were mapped to GO terms in the GO database (http://www.geneontology.org/) and significantly enriched GO terms were defined by hypergeometric test. KEGG as a public pathway‐related database could identify significantly enriched metabolic or signal transduction pathways in DEG. The calculating formula is the same as GO analysis.

### 2.5 The qRT‐PCR analysis

In three treatment groups (Diet I, Diet II, and Diet IV2), 37 differentially coexpressed genes (DCEG) were further analyzed to validate the RNA‐seq results by qRT‐PCR using CFX96 RT‐PCR Detection System (Bio‐Rad) with SYBR Green Kit (Promega). The extraction and determination of total RNA from the hepatic tissue were performed as in Section 2.4. cDNA was generated from DNase‐treated RNA by the FastQuant RT‐PCR Kit (with gDNase). The mixture contained 400 ng RNA, 2 μl Fast RT buffer (10×), 1 μl RT Enzyme Mix, 2 μl FQ‐RT Primer Mix, and 5 μl RNase‐Free ddH_2_O. The reaction conditions were 42°C for 15 min and 95°C for 3 min. The primers were designed according to Illumina sequencing data with Primer Premier 5. The reaction conditions were 95°C for 90 s followed by 40 cycles consisting of 95°C for 5 s, 60°C for 15 s, and 72°C for 20 s. The fluorescent flux was recorded, and the reaction continued at 72°C for 3 min. The dissolution rate was measured between 65 and 95°C. Each increase of 0.2°C was maintained for 1 s, and fluorescent flux was recorded. A dissociation curve was determined during PCR program to make sure that specific products were obtained in each run. The gene expression was analyzed by 2^−ΔΔCT^ method and normalized toward the mean of the reference gene (β‐actin). The stability of β‐actin was checked, which showed that ΔCt values among all treatments were less than 0.5.

### 2.6 The targeted quantitative proteomics based on mass spectrometry

Protein extraction and trypsin digestion. The sample was grinded in liquid nitrogen into cell powder and then transferred to a 5 ml centrifuge tube. After that, four volumes of lysis buffer (8 M urea, 1% Triton X‐100, 10 mM dithiothreitol, and 1% Protease Inhibitor Cocktail) were added to cell powder, followed by sonication three times on ice using a high‐intensity ultrasonic processor (Scientz). The remaining debris was removed by centrifugation at 20,000 g at 4°C for 10 min. Finally, the protein was precipitated with ice‐cold 20% TCA for 2 hr at −20°C. After centrifugation at 12,000 *g* 4°C for 10 min, the supernatant was discarded. The remaining precipitate was washed with cold acetone for three times. The protein was redissolved in 8 M urea, and the protein concentration was determined with BCA kit according to manufacturer's instructions. For digestion, the protein solution was reduced with 5 mM dithiothreitol for 30 min at 56°C and alkylated with 11 mM iodoacetamide for 15 min at room temperature in darkness. The protein sample was then diluted to urea concentration less than 2 M. Finally, trypsin was added at 1:50 trypsin‐to‐protein mass ratio for the first overnight digestion and 1:100 trypsin‐to‐protein mass ratio for the second 4 hr digestion.

LC‐MS/MS Analysis. The tryptic peptides were dissolved in 0.1% formic acid in water (solvent A), directly loaded onto a homemade reversed‐phase analytical column (15 cm length, 75 μm i.d.). The gradient was comprised of an increase from 6% to 23% solvent B (0.1% formic acid in 98% acetonitrile) over 38 min, 23% to 35% in 14 min and climbing to 80% in 4 min then holding at 80% for the last 4 min, all at a constant flow rate of 400 nl/min on an EASY‐nLC 1000 UPLC system. The peptides were subjected to NSI source followed by tandem mass spectrometry (MS/MS) in Q Exactive™ Plus (Thermo) coupled online to the UPLC. The electrospray voltage applied was 2.0 kV. The *m*/*z* scan range was from 350 to 1,000 for the full scan, and intact peptides were detected in the Orbitrap at a resolution of 35,000. Peptides were then selected for MS/MS using NCE setting as 27 and the fragments were detected in the Orbitrap at a resolution of 17,500. A data‐independent procedure alternated between one MS scan followed by 20 MS/MS scans. Automatic gain control (AGC) was set at 3E6 for full MS and 1E5 for MS/MS. The maximum IT was set at 20 ms for full MS and auto for MS/MS. The isolation window for MS/MS was set at 2.0 *m*/*z*. The resulting MS data were processed using Skyline (v.3.6). Peptide settings: enzyme was set as Trypsin [KR/P]; Max missed cleavage was set as 2. The peptide length was set as 8–25, variable modification was set as carbamidomethyl on Cys and oxidation on Met, and max variable modifications were set as 3. Transition settings: precursor charges were set as 2 and 3, ion charges were set as 1 and 2, and ion types were set as *b*, *y*, and *p*. The product ions were set as from ion 3 to last ion, and the ion match tolerance was set as 0.02 Da.

### 2.7 Statistical analysis

Based on obtained data, the mean and standard deviation in each group were calculated, and one‐way analysis of variance was conducted by using SPSS 19.0 software. *p* < .05 indicates that the two groups are different, and *p* < .01 indicates that the two groups have significant difference.

## 3 RESULTS

### 3.1 The changes in physiological indexes in mice

#### 3.1.1 The ameliorating effect of FRR on hepatic pathology

**FIGURE 1:**
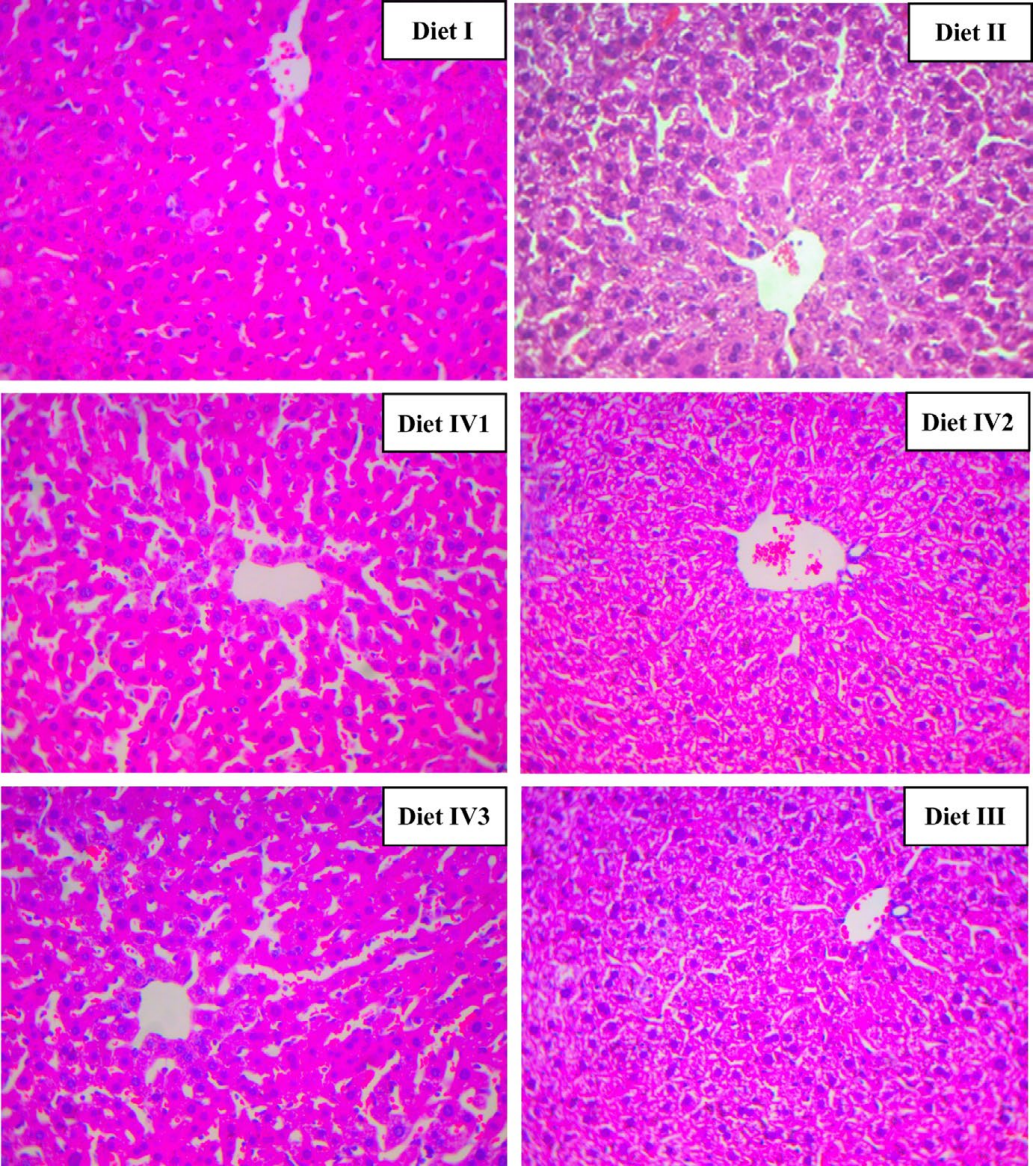
The pathological analysis of KM mice liver in different treatments on day 30, including control (Diet I), model (Diet II), positive drug (Diet III), 25% FRR (Diet IV1), 50% FRR (Diet IV2), and 100% FRR (Diet IV3)

On day 30, the hepatic pathological changes in mice were analyzed by HE staining in different groups. In Diet I group, hepatic lobules were intact, with radially arranged hepatic cords around central vein and no obvious lipid infiltration. In Diet II group, hepatic cords were loosely arranged, and hepatocytes with medium microvesicular steatosis were swollen. In Diet III and Diet IV2 groups, hepatic steatosis was significantly relieved, but no obvious effect in Diet IV1 and Diet IV3 (Figure 1).

#### 3.1.2 The modulation of FRR on blood lipids

**FIGURE 2:**
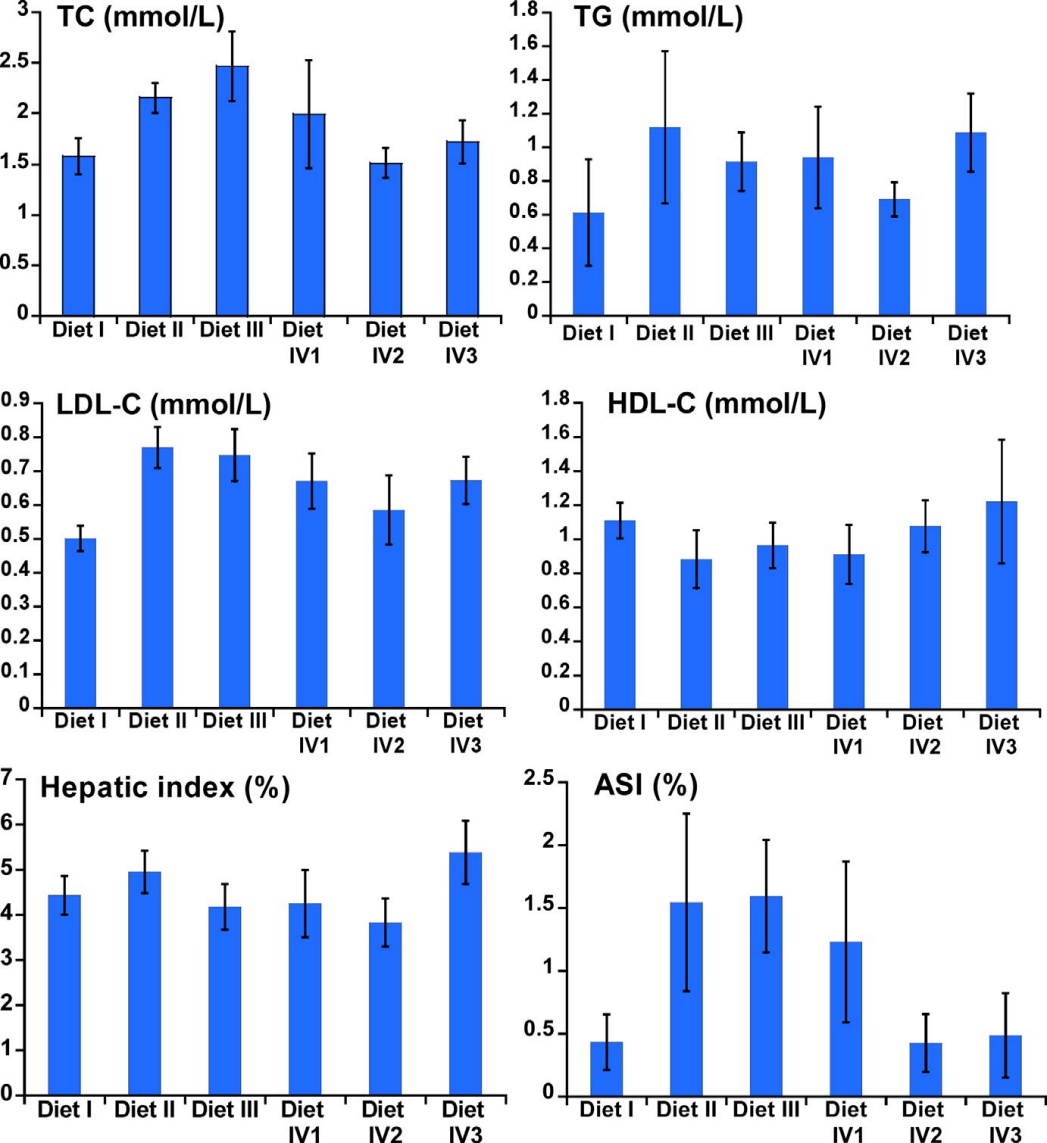
The modulating effects of FRR on blood lipids of KM mice in different treatment groups on day 30 (TC, TG, LDL‐C, HDL‐C, hepatic index, and ASI)

The blood lipids in different treatments were measured on day 30. In Diet II group, TC (2.15 mmol/L), TG (1.12 mmol/L), LDL‐C (0.77 mmol/L), hepatic index (4.95 mmol/L), and ASI (1.55 mmol/L) in serum were significantly increased (*p* < .05) and HDL‐C (0.88 mmol/L) was lower (*p* < .05) compared with Diet I group. In Diet III group, blood lipid level in serum was close to Diet I group, and hepatic index (4.18 mmol/L) was lower than Diet I (*p* < .05). In Diet IV2 group, TC, TG, LDL‐C, HDL‐C, and ASI in serum were regulated to control level, and hepatic index (3.83 mmol/L) was significantly lower than Diet I (Figure 2, *p* < .05).

### 3.2 The transcriptomic analysis for hepatic tissue of mice by RNA‐seq

#### 3.2.1 Mapping of RNA‐Seq reads and functional annotation of the hepatic transcriptome

At present, 9 cDNA libraries were created by Illumina sequencing (Table 1). Every treatment had three replicates. Q20 was 96.08%–96.43%; Q50 was 89.48%–90.21%.

**TABLE 1:** Summary of RNA data processing from mice livers

Sample no.	Treatment^b^	HQ^a^ clean data After filter (bp)	Q20 (%)	Q30 (%)
C1	Diet I	4,511,773,540	4,342,615,997 (96.25%)	4,052,223,237 (89.81%)
C2	Diet I	4,497,119,645	4,334,192,745 (96.38%)	4,052,232,196 (90.11%)
C3	Diet I	4,549,136,921	4,373,287,541 (96.13%)	4,074,538,721 (89.58%)
G1	Diet II	3,623,642,042	3,481,762,457 (96.08%)	3,242,522,426 (89.48%)
G2	Diet II	5,065,168,516	4,877,705,815 (96.30%)	4,552,634,623 (89.88%)
G3	Diet II	4,765,874,891	4,586,379,652 (96.23%)	4,276,542,918 (89.73%)
C50‐1	Diet IV2	4,480,854,725	4,309,003,781 (96.16%)	4,014,014,812 (89.58%)
C50‐2	Diet IV2	4,508,765,858	4,347,595,127 (96.43%)	4,067,490,011 (90.21%)
C50‐3	Diet IV2	4,689,900,892	4,513,364,104 (96.24%)	4,208,659,713 (89.74%)

^a^ High quality.

^b^ Three replicates for every treatment.

**FIGURE 3:**
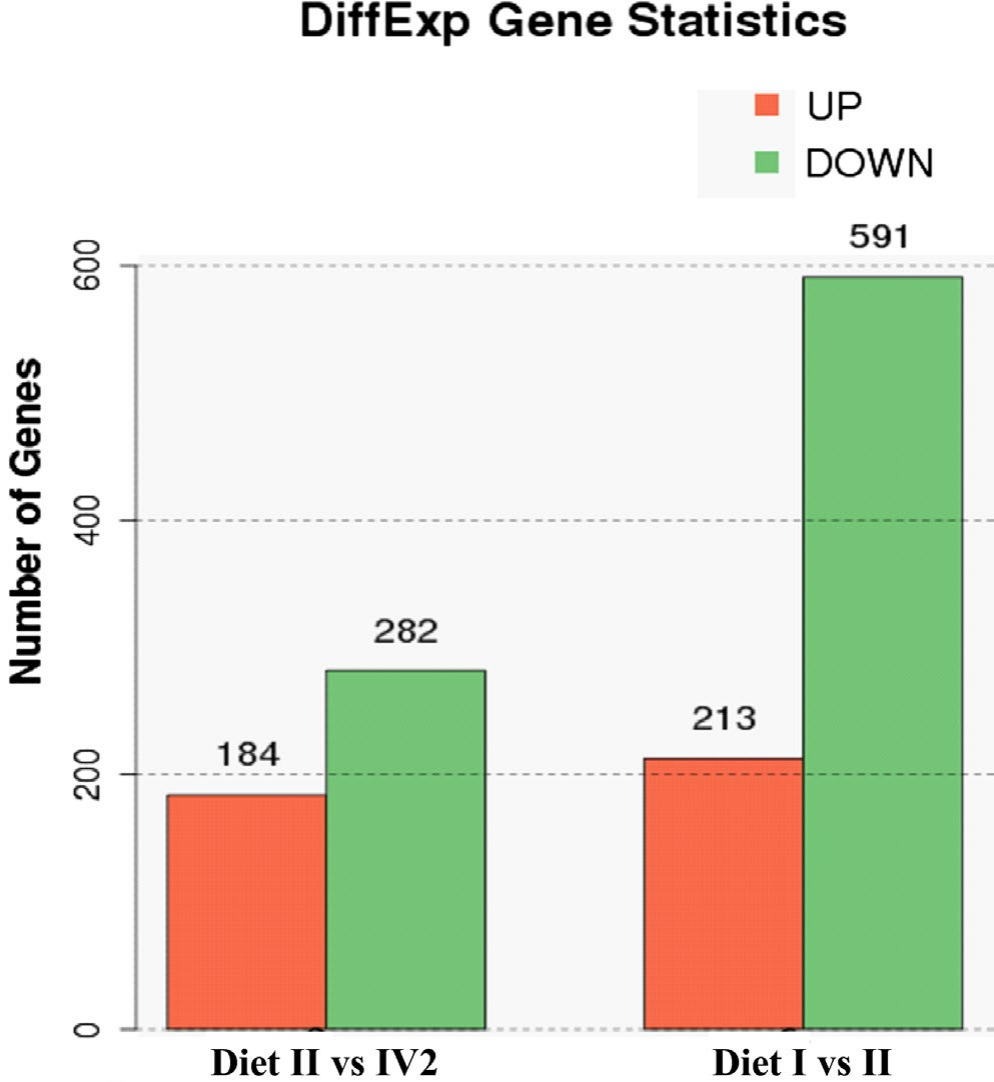
Bioinformatic analysis of RNA‐seq data in KM mice. The number of DEG between Diet I and Diet II, and Diet II and Diet IV2 groups

A pairwise comparison was performed by using Diet I as the control, and Diet II and Diet IV as the treatments. A total of 1,270 genes were identified as DEG and used for further analysis. The criteria that were used to determine significantly up‐ or downregulated genes were a twofold or greater change in expression, *p*‐value < .05 and false discovery rate (FDR) < 0.05. As a result, 804 genes (213 upregulated and 591 downregulated) displayed the changes in expression between Diet I and Diet II, 466 genes (184 upregulated and 282 downregulated) between Diet II and Diet IV2 (Figure 3).

#### 3.2.2 The identification of DCEG

To reveal the molecular mechanism associated with lipid metabolism, 623 genes in the liver for three dietary treatments were evaluated by RNA‐seq. Three hundred eighty‐eight genes were found in Diet I and Diet II groups; compared with Diet I, 110 were upregulated and 278 were downregulated in Diet II. Two hundred thirty‐five genes were found in Diet II and Diet IV2 groups; compared with Diet II, 90 were upregulated and 145 genes were downregulated in Diet IV2. From these 623 genes, only 37 DCEG were found in all three Diet I, Diet II, and Diet IV2 groups (Table 2), 17 genes were upregulated in Diet II and then downregulated in Diet IV2, 16 genes were downregulated in Diet II and then upregulated in Diet IV2, one gene kept the upregulation and two genes downregulation in Diet II and IV2 groups, and one gene was only detected in Diet II and Diet IV2 groups. These DCEG were participated in lipid metabolism directly or indirectly (Table 2).

**TABLE 2:** The DCEG and enriched KEGG pathways from hepatic tissue in different treatments

KEGG pathway and ID	Genes symbol	FPKM^a^ (Diet I/II/IV2)
Fatty acids metabolism		
Glycerophospholipid metabolism, K14156	Chka	19.50/76.46/11.71
Fatty acid elongation, K10248	Elovl3	91.02/650.68/81.43
Lipid metabolism, K04452	Ddit3	3.00/17.73/4.46
Fatty acid oxidation and biosynthesis, K07202	Ppargc1a	3.47/0.375/1.6
Fatty acid metabolism, K03460	Slco1a4	17.17/4.415/20.30
Fatty acid biosynthesis and adipocyte differentiation, K19518	Trib3	7.175/0.71/9.73
Cholesterol metabolism, K05062	Lepr	1.61/0.22/1.50
Arachidonic acid and linoleic acid metabolism, K07413	Cyp2c39	155.71/33.3/155.80
Fatty acid and cholesterol metabolism, K00489	Cyp7a1	35.57/4.26/23.70
Arachidonic acid and linoleic acid metabolism, K07413	Cyp2c37	37.86/9.30/67.25
Arachidonic acid and linoleic acid metabolism, K07413	Cyp2c50	237.32/90.07/251.88
Fatty acid metabolism, K17687	Cyp4a10	179.14/21.27/1209.86
Arachidonic acid and linoleic acid metabolism, K07413	Cyp2c54	140.85/28.20/194.12
Lipid and lipoprotein metabolism, K15728	Lpin1	49.22/3.20/9.72
Fatty acid and arachidonic acid metabolism, K17687	Cyp4a32	172.03/22.95/259.50
Unsaturated fatty acid biosynthesis, K01068	Acot3	0.49/2.15/21.84
Fatty acid biosynthesis, K00665	Fasn	—/33.35/13.18
Other related metabolism and signaling pathways		
Atherosclerosis and nuclear‐initiated steroid signaling, K04079	Hsp90aa1	30.73/144.03/23.29
Atherosclerosis and fluid shear stress, K05692	Actb	192.48/531.34/137.43
Atherosclerosis and fluid shear stress, K05692	Actg1	94.89/429.67/58.55
Transcriptional activation, cAMP signaling pathway, K18435	Sox9	1.13/6.17/0.55
Cell differentiation, TGF‐β signaling pathway, K04661	Fst	2.02/8.07/0.48
Apoptosis and cytoskeleton, K07374	Tuba4a	44.43/148.68/63.37
Tumorigenesis, K09485	Efna1	22.21/70.33/18.85
Apoptosis, K05462	Hsph1	17.96/73.49/9.95
Brown fat differentiation, K07375	Tubb4b	51.27/154.25/43.48
Metal ions and cell matrix, K19721	Col27a1	0.88/5.36/2.19
Cytoskeleton component, K07375	Tubb2a	41.81/227.45/16.45
Apoptosis and signaling pathway, K04459	Dusp6	8.94/93.47/9.83
Cell adhesion and cytokine signaling pathway, K04198	Tnfrsf12a	8.24/96.16/11.59
Apoptosis, MAPK and P53 signaling pathway, K04402	Gadd45a	1.36/20.12/2.16
Calcium channels and cGMP signaling pathway, K04198	Ednrb	4.36/1.31/3.74
Nucleotide catabolism, K00757	Upp2	131.25/33.65/101.88
Calcium channels and pancreatic secretion, K04515	Camk2b	3.19/1.86/5.85
Formic acid degradation, K00789	Mat1a	2,221.69/439.86/1687.62
Cell adhesion, antigen presentation, endocytosis, K06751	H2‐Q7	45.31/10.96/35.08
Phagolysosome and peroxidase activity, K10789	Mpo	15.26/0.79/0.06

^a^ FPKM is fragments per kilobase of exon model per Million mapped reads. The fold change in down‐ or upregulated genes were log2 (Fc) ≥ 2, *p* < .05. "—" represents the gene was not detected.

**FIGURE 4:**
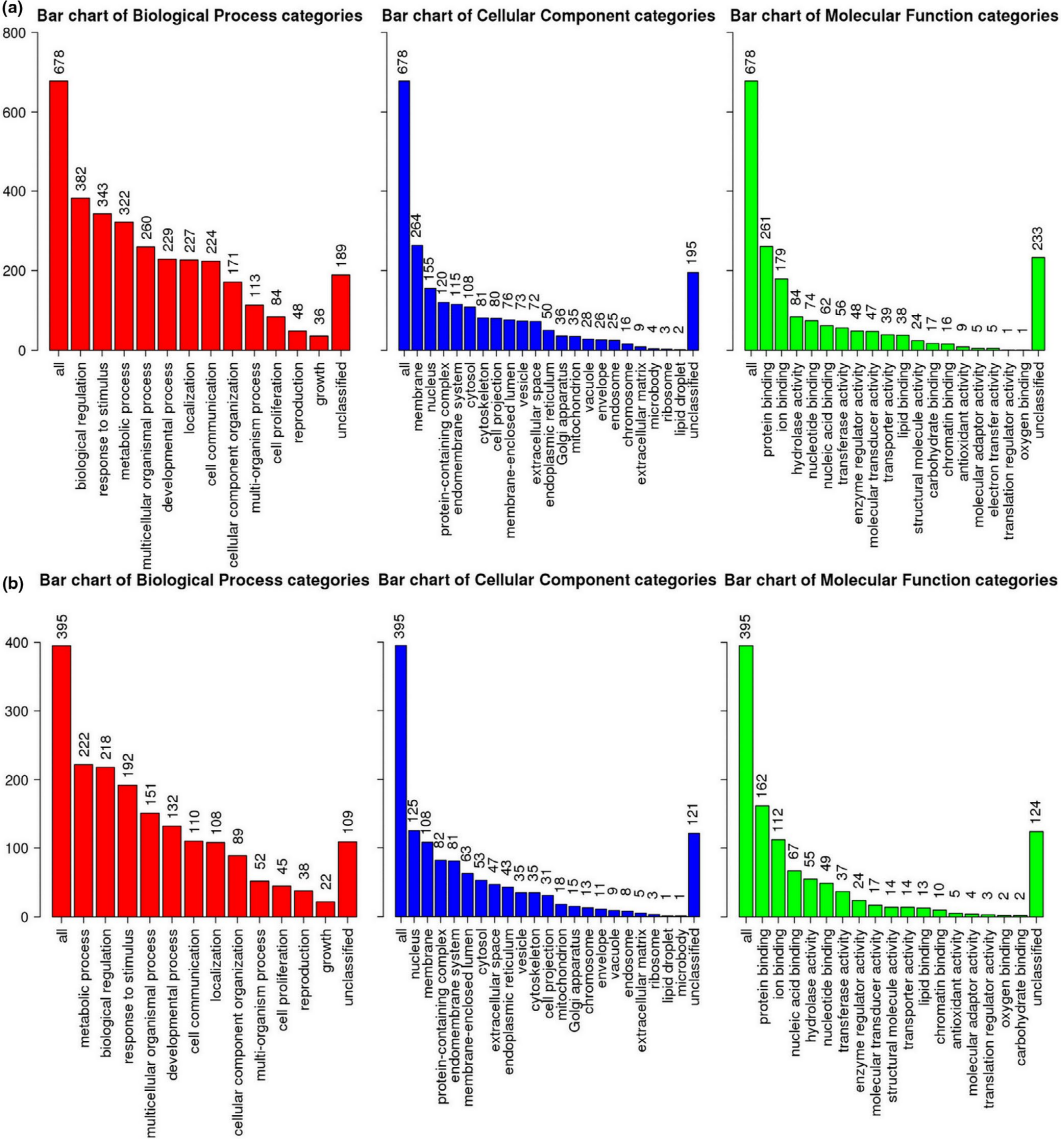
GO annotations of DEG in liver tissue between the groups of Diet I and Diet II (a), and Diet II and Diet IV2 (b)

**FIGURE 5:**
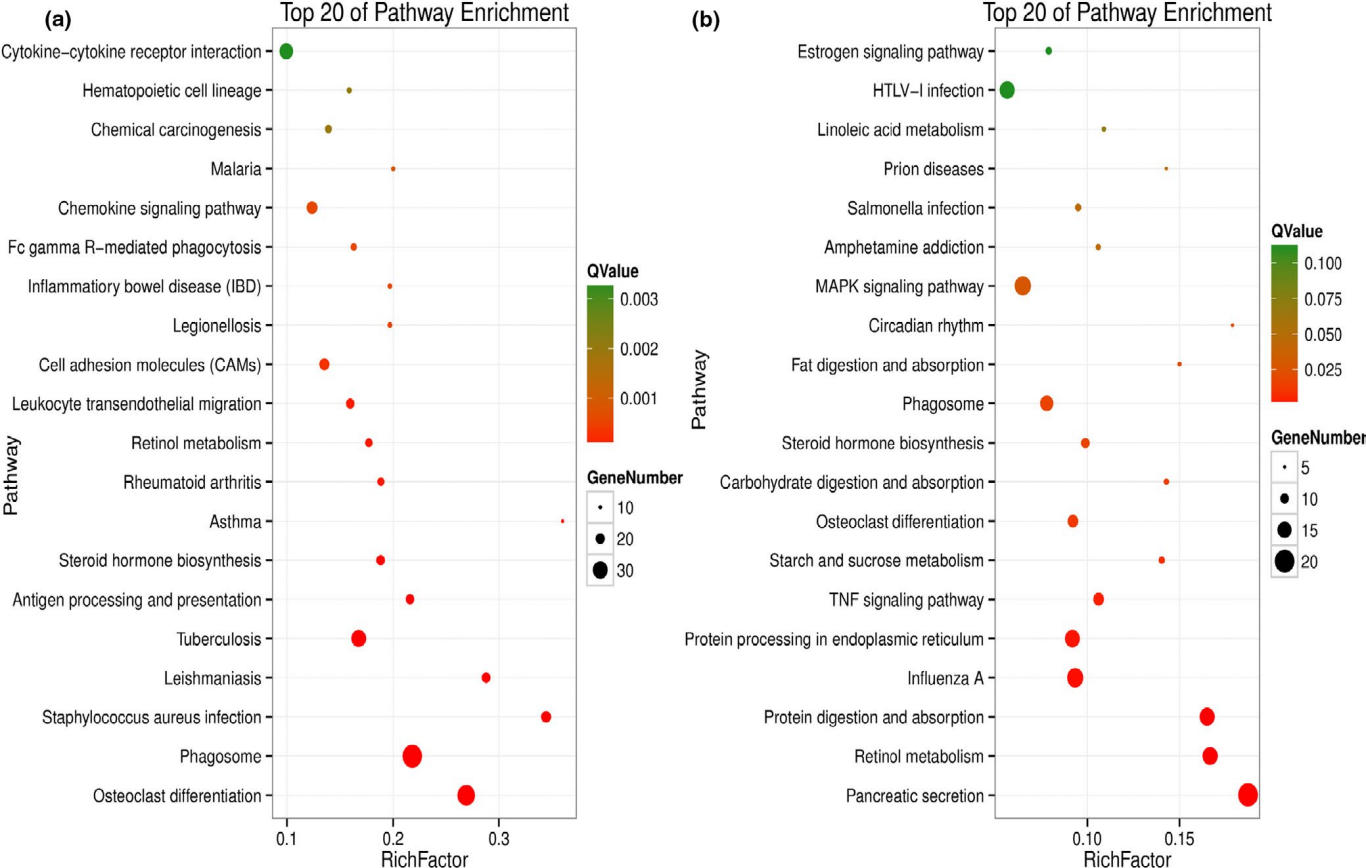
The top 20 of KEGG pathway of DEG in liver between the groups of Diet I and Diet II (a), and Diet II and Diet IV2 (b). RichFactor denotes the ratio of gene numbers in one pathway and all pathways. *Q*‐value is tested; *P*‐value ranging from 0 to 1

**FIGURE 6:**
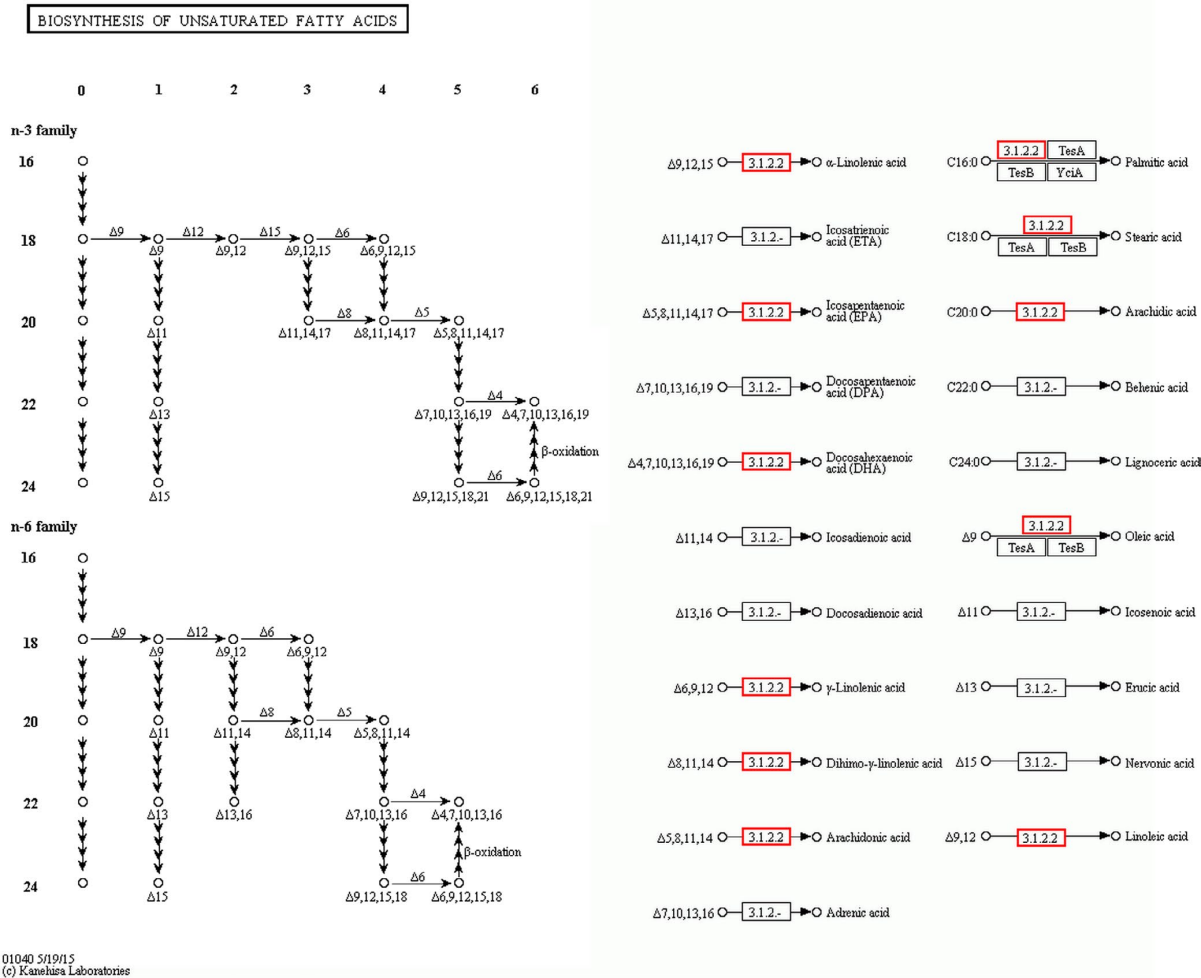
The gene (Acot3) associated with biosynthesis of unsaturated fatty acids was annotated by KEGG. Red denotes Acot3 in mice fed Diet IV2 was upregulated compared to Diet II group

To characterize the functional consequences of genes in liver, a Gene Ontology (GO) functional enrichment and a KEGG pathway analysis of DEG were performed for each treatment. These genes were mapped to GO databases and classified into three major functional categories such as biological process, cellular component, and molecular function (Figure 4a,b). The GO enrichment analysis showed that a large number of biological processes were significantly altered (*q*‐value < 0.05) between transcriptomes of three treatment groups (Diet I vs. II and II vs. IV2), respectively. To identify the active biological pathways of liver in different treatments, the annotated coding sequences of differential genes were mapped to KEGG database. Comparing Diet I and Diet II groups, twenty KEGG terms were significantly enriched (*q*‐value < 0.05, Figure 5a). The twenty KEGG terms between the groups of Diet II and IV2 were also significantly enriched (Figure 5b). The bigger the RichFactor is, the higher the enrichment is. If *Q*‐value is closer to zero, enrichment is more significant. From Figure 6b, it is seen that the pathway of fat digestion and absorption is significantly enriched.

### 3.3 The qRT‐PCR validation of DCEG

To confirm the reliability of the gene expression profiles generated by RNA‐seq analysis, qRT‐PCR was used to examine the expression levels of 37 DCEG shown in Table 2 in hepatic mRNA samples. Nine DCEG that had different expression profiles between RNA‐seq analysis and qRT‐PCR were discarded from further analysis as ambiguous. For 28 of these genes, the expression patterns detected by qRT‐PCR were matched with those genes by RNA‐seq analysis (Table 3). They might interact with each other to regulate lipid metabolism by specific signaling pathways, which could commonly maintain metabolic homeostasis (Tables 2 and 3).

**TABLE 3:** The validation of DCEG by qRT‐PCR

Genes name	Primer (5′−3′, F/R)	2^−ΔΔCт^		
		Diet I	Diet II	Diet IV2
Hsp90aa1	AGGACCGAACCCTGACCAT/GCTCATCGTCGTTATGCTTCG	1.00 ± 0.05	1.12 ± 0.03	0.43 ± 0.03
Chka	AACTCTTTGGCATCTTTCCC/TCAAGAGGCAGGTTGTAAGAGA	1.00 ± 0.06	1.30 ± 0.03	0.58 ± 0.05
Elovl3	CATCATCCTGCGTAAGCGTC/CGAAGGCACTTTGTTCTTGTATC	1.00 ± 0.05	14.93 ± 0.03	0.10 ± 0.05
Ppargc1a	AAAACAGGAACAGCAGCAGAG/CAGAGGAAGAGATAAAGTTGTTGG	1.00 ± 0.04	0.10 ± 0.03	0.35 ± 0.06
Slco1a4	GGGGTTGCCTGCTGCTCTA/GTTTTCCGTTCTCCATCATTC	1.00 ± 0.05	0.08 ± 0.05	2.13 ± 0.04
Trib3	AGATGCCTGCGTGATGACTG/CCGCTTTGCCAGAGTAGGAT	1.00 ± 0.05	0.30 ± 0.04	0.77 ± 0.05
Lepr	GCCAAACTCAACTACGCTCTTC/ATTGAAGCGGAAATGGTGC	1.00 ± 0.09	0.07 ± 0.10	0.94 ± 0.07
Cyp2c39	GATTCATCAACCTTGTCCCTAAC/GGTGGTCAGGAATAGAAACAGC	1.00 ± 0.05	0.54 ± 0.04	1.98 ± 0.03
Cyp7a1	TTGATTCCATACCTGGGCTGT/TGACAGGGAGTTTGTGATGAAG	1.00 ± 0.06	0.11 ± 0.05	0.35 ± 0.03
Cyp2c37	GACAACAAGCACAACACTGAGAT/TTGGGGAACTCCGTGCTGT	1.00 ± 0.08	0.57 ± 0.03	1.77 ± 0.03
Cyp2c50	ACATCTGCCAATCCTTCACC/ATTCCGCAGAGTCGTGAGTG	1.00 ± 0.05	0.45 ± 0.03	1.10 ± 0.04
Cyp4a10	TCTCTGCTCTAAGCCCAACC/CTGGAAAGCCTTGAGTAGCC	1.00 ± 0.06	0.10 ± 0.03	11.39 ± 0.03
Cyp2c54	GTTTGACCCTGGGCACTTTCT/CATATCCGTTTCCCTGTTGAG	1.00 ± 0.06	0.39 ± 0.10	1.37 ± 0.04
Acot3	CCTACCTGCTCAGTCACCCTC/AGTTTCCGCCGATGTTGG	1.00 ± 0.06	6.20 ± 0.05	99.69 ± 0.07
Lpin1	GCTTCGGCAAGATGGGTG/AAATGCTTCTCCGTTGTCTCC	1.00 ± 0.05	0.31 ± 0.04	0.12 ± 0.05
Sox9	GTCCCAGCGAACGCACAT/TGGTCAGCGTAGTCGTATTGC	1.00 ± 0.09	1.35 ± 0.05	0.90 ± 0.06
Tuba4a	GACCTGGAGCCTACTGTAATCG/CAGCAGAGAGGTGAAGCCAGA	1.00 ± 0.05	4.28 ± 0.03	2.30`±0.06
Efna1	ACAGTTCAAATCCCAAGTTCCG/GCCACAGAGTCGTCCTCGTAA	1.00 ± 0.05	0.83 ± 0.03	0.88 ± 0.04
Hsph1	ACTTGGTATGGCAGTTAGGGAG/CCCTTCCTCATACAGCCAGTC	1.00 ± 0.06	1.05 ± 0.04	0.39 ± 0.03
Col27a1	CCCACAGCCAATGTTCTTCC/TGCGGATGGCAAAGAGG	1.00 ± 0.05	2.25 ± 0.03	1.58 ± 0.03
Tubb2a	TGGTGAGGAAGGAGTCTGAAAG/GGGCTCCACCACAGTATCAG	1.00 ± 0.07	5.51 ± 0.04	0.76 ± 0.04
Ednrb	TGGTGGCTGTTCAGTTTCT/GCATACCGCTCTTCTTCCTG	1.00 ± 0.04	0.21 ± 0.03	1.08 ± 0.04
Upp2	GGTTGGAGGGAGATGGAGA/TGACTGACGGAGAGCACG	1.00 ± 0.06	0.10 ± 0.03	0.54 ± 0.03
Camk2b	AGCATTCCAACATTGTACGC/CTCCCCACCAGTGACCAGAT	1.00 ± 0.25	0.13 ± 0.32	1.95 ± 0.26
Mat1a	CGTGCTCGCTCACAAACTCA/CAATAACAAACCGCCCACTT	1.00 ± 0.05	0.19 ± 0.04	0.96 ± 0.03
H2‐Q7	GCAGTCGCTCCGCAGATA/TTCCCAAGAGGCACCACC	1.00 ± 0.05	0.10 ± 0.04	0.42 ± 0.03
Mpo	ATCGCCAATGTCTTCACC/GCTCAAATAGTCGCTCCCG	1.00 ± 0.05	0.003 ± 0.13	0.01 ± 0.10
Tubb4b	GCTAAATGCTGACCTGCGG/TGTTCGTCCACCTCCTTCATAG	1.00 ± 0.05	2.76 ± 0.03	0.99 ± 0.04

Note

2^−ΔΔCт^ represents the relative quantity of gene. F is forward primer, and R is reversed primer.

### 3.4 Targeted‐protein identification

To verify the protein expression of these DCEG, targeted proteins in liver tissues were analyzed by mass spectrometry. In total, 10 DCEP were detected by targeted quantitative proteomic analysis, which were related to lipid metabolism directly or indirectly (Table 4). Among them, Mat1a, Fasn, Cyp7a1, Cyp2c39, Lpin1, Mpo, Hsph1, and Cyp4a10 were downregulated 0.1‐fold in Diet II and then upregulated in Diet IV2. Cyp2c54 and Hsp90aa1 were always downregulated in Diet II and Diet IV2 groups. Quantitative ratio over 1.3 was considered as upregulation, while less than 1/1.3 (~0.77) was considered as downregulation.

**TABLE 4:** The expression of DCEP by targeted quantitative proteomic analysis

Gene name	Protein accession	Protein description	MW (kDa)	Diet II/I	Diet IV2/II
Mat1a	Q91X83	Methionine adenosyltransferase 1a	43.51	0.12	2.57
Fasn	P19096	Fatty acid synthase	272.43	0.11	3.36
Cyp7a1	Q64505	Cholesterol 7‐α‐monooxygenase	57.26	0.002	49.59
Mpo	P11247	Myeloperoxidase	81.18	0.06	4.38
Cyp2c54	Q6XVG2	Cytochrome P450 2C54	55.86	0.24	1.13
Cyp2c39	P56656	Cytochrome P450 2C39	55.83	0.03	2.04
Hsp90aa1	P07901	Heat‐shock protein 90‐alpha	84.79	0.20	1.16
Hsph1	Q61699	Heat‐shock protein 105 kDa	96.41	0.24	3.22
Lpin1	Q91ZP3	LPIN1	102	0.001	44.10
Cyp4a10	O88833	Cytochrome P450 4a10	58.33	0.09	6.86

## 4 DISCUSSION

In view of rich nutrients and bioactive properties, FRR has been considered as a dietary supplement that could ameliorate the dyslipidemia and finally alleviate the risk of AS (Xu et al., 2019). However, detailed mechanisms are still unclear. It is well known that HLP is primary factor in the pathophysiological process forming AS (Ross & Harker, 1976). At present, we investigate the protection effects and molecular mechanism of FRR juice on diet‐induced hyperlipidemic mice and explore the potential anti‐atherosclerosis.

The basic physiological–biochemical indexes had significant differences indifferent treatment groups in mice. Compared with control group (Diet I), TC, TG, LDL, and AS index of KM mice fed Diet II were significantly increased in serum; meanwhile, HDL level was significantly decreased. Lipoproteins are closely associated with atherosclerotic vascular process. Elevated HDL and apolipoprotein AI (apo AI) in plasma suggest a low probability of AS, but elevated LDL and apo B indicate an increased risk of AS (Gille, D'Andrea, Tortorici, Hartel, & Wright, 2018; Nayak et al., 2016). In Diet IV2 group, hepatic steatosis of KM mice was substantially attenuated, which had similar change with Diet III group (Figure 1). The TC, TG, LDL‐C, HDL‐C, and ASI in serum were also alleviated to control level; especially, hepatic index was significantly lower than control group (Figure 2). For Diet IV group, blood lipids of mice fed Diet IV2 were regulated to optimal level comparing to Diet IV1 and Diet IV3. From these findings, it was concluded that supplementation of appropriate FRR could modulate the blood lipids in normal level. It is notable that many medicinal and edible plants contain a large amount of bioactive products, and they could play an important role in regulating the body metabolism by multifunction or synergistic effect (Qin & Xu, 1998).

Transcriptome is defined as complete set of expressed RNA transcript in a cell or tissue, which can provide understanding for cellular activities including the growth, development, and metabolism in organisms, particularly in exploring potential mechanism of nutrients (Ogłuszka et al., 2017; Pertea et al., 2015). RNA sequencing is an effective method that could serve as a platform for future investigations. At present, totally 1,273 genes in liver were generated, of which 623 genes in three dietary treatments (Diet I, Diet II, and Diet IV2) were annotated against KEGG database. To characterize the functions of genes, these DEG in three treatments were analyzed by GO function (Figure 4) and KEGG pathway (Figure 5). Pathway enrichment analysis indicated that lipid metabolism was significantly changed in mice fed Diet II and Diet IV2, which highlighted the importance of liver with variable rates of lipid metabolism. Further analysis, 37 DCEG were screened out in three treatment groups (Table 2). These genes were involved in lipid metabolism and signal transduction, which showed that biological processes in HLP mice were significantly altered by FRR. To validate the reproducibility and accuracy of RNA‐seq analysis, relative mRNA levels of 37 DCEG were determined by qRT‐PCR, of which 28 genes were matched with those detected by RNA‐seq analysis (Table 3). Their fold changes in genes expression ratios between the RNA‐seq and qRT‐PCR analyses were positively correlated, which verified the reliability of RNA‐seq analysis. These results also showed that qRT‐PCR expression patterns of genes were consistent with those of RNA‐seq. By data analysis, it was found that these DCEG played important roles in regulating lipid metabolism, mainly including biosynthesis of bile acids (BAs) and steroids, fatty acid metabolism, and lipid peroxides (LPOs) production.

The expression of 10 genes was decreased in Diet II group, but returned to control level or obviously increased in Diet IV2 group, including *Cyp2c39*, *Cyp7a1*, *Cyp2c37*, *Cyp2c50*, *Cyp4a10*, *Cyp2c54*, *Ppargc1a*, *Trib3*, *Lepr*, and *Mat1a*. Under the FRR action, upregulated expression of six genes in cytochrome P450 (Cyp) family might promote the biosynthesis of BAs and steroids by increasing the cholesterol catabolism (Kandel et al., 2016; Kuncharoenwirat & Jarukamjorn, 2019; Russo et al., 2018; Vangaveti, Jansen, Kennedy, & Malabu, 2016). They might also regulate lipid metabolism by the signaling pathway of peroxisome proliferator‐activated receptor (PPAR) (Rigano, Sirignano, & Taglialatela‐Scafati, 2017). PPAR as key nuclear factor could regulate the function of HDL and RCT, and anti‐atherosclerotic function of HDL was mainly conducted by RCT (Ikhlef, Berrougui, Kamtchueng Simo, Zerif, & Khalil, 2017; Marques et al., 2018; März et al., 2017). PPARα is also a key therapeutic target for HLP (Araki et al., 2018). PPAR signaling pathway might play an important role in promoting RCT. Researches indicated that fibrates as hypolipidemic agents could activate PPARα pathway and increase Cyp content, and resulting oxysterol generation may mediate the RCT by regulating the expression of proteins involved in lipid metabolism (Cizkova, 2018; Giampietro, Ammazzalorso, Amoroso, & De Filippis, 2019). Cyp could restrain cholesterol accumulation and AS formation by regulating the proliferation and migration of vascular smooth muscle cells (Bennett, Jørgensen, Clarke, Bennett, & Mallat, 2016; Jefcoate & Larsen, 2019). *Ppargc1a* as a target could also regulate lipid metabolism. It was found that miR‐30b‐5p might regulate lipid metabolism by targeting the *Ppargc1a* in hepatocellular carcinoma Huh‐7 cells (Zhang et al., 2020). *Trib3* and *Lepr* were both associated with the control of fatty acid synthesis besides for regulating TG, TC, and HDL‐C levels in plasma (Angyal & Kiss‐Toth, 2012; Domínguez‐Reyes et al., 2015). *Mat1a* plays an important role in amino acid metabolism and formic acid degradation, especially modulating the effect of fatty acids on plasma homocysteine concentrations (Yang et al., 2019; Poursoleiman et al., 2018). *Mat1a* is also necessary for normal VLDL assembly and lipid homeostasis in plasma (Cano et al., 2011).

The expression of four genes (*Tuba4a*, *Tubb2a*, *Tubb4b*, *and Elovl3*) was increased in Diet II group, but decreased in Diet IV2 group. The lipid composition in membrane could regulate tubulin interaction with mitochondrial voltage‐dependent anion channel (VDAC). The electron transport chain is major cellular source for mitochondrial reactive oxygen species (ROS). αβ‐Tubulin heterodimers could inhibit VDAC conductance in lipid bilayers, thus decreasing mitochondrial metabolism (Fang & Maldonado, 2018; Rostovtseva, Gurnev, Chen, & Bezrukov, 2012). It was also found that the agonist pemafibrate could increase the expression of mitochondrial marker *Elovl3* involved in fatty acids oxidation (Araki et al., 2018).

The expression of two genes (*Lpin1* and *Mpo)* was both decreased in Diet II and Diet II IV2 groups. *Lpin1* could regulate the metabolism of TG and phospholipid, and catabolism of fatty acids, thus preventing excessive fat accumulation (Reue & Brindley, 2008). Lpin1 overexpression in response to nutritional stress might inhibit the LDL synthesis (Assaily et al., 2011; Bi, Jiang, & Zhou, 2015). Mpo secreted by activated phagocytes has been implicated in the development of HLP and atherosclerotic lesions. Mpo can catalyze oxidation reactions and oxidize LDL by using hydrogen peroxide and halide ions to give hypochlorous (HOCl) or hypobromous (HOBr) acids and tyrosyl radical (Castellani, Chang, Wang, Lusis, & Reynolds, 2006; Podrez, Abu‐Soud, & Hazen, 2000). Mpo could activate NF‐κB in RCT by converting HDL into proinflammatory particle. Mpo could be affected by inflammation or glycation‐related factors, especially esterification or unesterification of lipids, which might change the HDL function (Ertek, 2018).

The increase in *Acot3* and decrease in *Chka* both appeared in Diet IV2 group. *Acot3* could promote the biosynthesis of polyunsaturated fatty acids (PUFAs); ω‐3 and ω‐6 PUFAs play important role in regulating the blood lipids and preventing the AS (Figure 6). The finding was consistent with previous data. Accumulating evidences also concluded that FRR could attenuate the risk of AS (Goldstein & Brown, 2009; Olsson et al., 2001), because various kinds of bioactive substances in FRR could regulate lipid metabolism by synergistic effect. *Chka* is the first enzyme in the CDP‐choline pathway, which has been implicated in phospholipid metabolism. Its overexpression in several human tumors has been proposed as a novel cancer drug target (Lacal & Campos, 2015). It was seen that lower expression of Chka was beneficial to maintain lipid metabolism under FRR action.

The protein expression corresponding to DCEG was further analyzed by targeted quantitative proteomic analysis. Only 10 proteins were exactly verified, all of them were downregulated in mice fed Diet II and upregulated in Diet IV2 group (Table 4). Among of them, expressive patterns of six proteins (Cyp2C39, Cyp4a10, Cyp7a1, Cyp2C54, Mpo, and Mat1a) were parallel with qRT‐PCR. These data show that mRNA abundance of specific gene was not necessarily a linear relationship with protein expression, because gene expression was regulated at different levels, especially during transcription and translation, which would affect the protein expression (Gautier et al., 2012; Spriggs, Bushell, & Willis, 2010). The degradation of mRNA and proteins, and protein modification may also lead to this inconformity. Furthermore, the abundance of some proteins might be too low to be detected by targeted quantitative proteomics.

In a word, FRR juice could ameliorate the blood lipids and maintain metabolic balance by regulating the expression of these DCEG and DCEP in mice. Despite different expression patterns at molecular level, they jointly regulated lipid metabolism and normalize the blood lipids in mice. In our experiments, HDL level in mice fed Diet IV2 was increased significantly, and expression of genes and proteins involved in PPAR signaling pathway was also altered. It can be seen that FRR juice could regulate the function of HDL or RCT by PPAR signaling pathway, thus maintaining normal level of blood lipids in mice, which might be important drug targets for AS. This would be very beneficial to explore anti‐atherosclerotic mechanism of FRR and provide theoretical basis for application of FRR in AS.

## 5 CONCLUSIONS

Under the FRR action, the TC, TG, LDL, HDL, and ASI in serum were regulated to control level. Thirty‐seven DCEG in each group were obtained by RNA‐seq analysis. Relative mRNA levels of 28 genes determined by qRT‐PCR were matched with those detected by RNA‐seq analysis. Ten DCEP were verified by targeted quantitative proteomic analysis, but expressive patterns of only six proteins were correlated with qRT‐PCR data. These DCEG and DCEP played important roles in regulating lipid metabolism, mainly including biosynthesis of BAs and steroids, fatty acid metabolism, and LPO production. They might cooperatively regulate the function of HDL or RCT by PPAR signaling pathway under the action of FRR juice.

## ETHICAL APPROVAL

The study's protocols and procedures were ethically reviewed and approved by the Institutional Animal Care and Use Committee (IACUC) of Guizhou Medical University of China. The authors agree with the paper publication. The data and materials in the study are shared and available. The authors declare that they have no any conflict of interests.
